# Potential cancer-related role of circadian gene *TIMELESS* suggested by expression profiling and *in vitro* analyses

**DOI:** 10.1186/1471-2407-13-498

**Published:** 2013-10-25

**Authors:** Yingying Mao, Alan Fu, Derek Leaderer, Tongzhang Zheng, Kun Chen, Yong Zhu

**Affiliations:** 1 Department of Epidemiology and Health Statistics, Zhejiang University School of Public Health, Hangzhou, Zhejiang Province, China; 2 Department of Environmental Health Sciences, Yale School of Public Health, New Haven, CT 06520, USA

**Keywords:** *TIMELESS*, Circadian gene, Cell cycle, Tumorigenesis, Expression profiling

## Abstract

**Background:**

The circadian clock and cell cycle are two global regulatory systems that have pervasive behavioral and physiological effects on eukaryotic cells, and both play a role in cancer development. Recent studies have indicated that the circadian and cell cycle regulator, TIMELESS, may serve as a molecular bridge between these two regulatory systems.

**Methods:**

To assess the role of TIMELESS in tumorigenesis, we analyzed TIMELESS expression data from publically accessible online databases. A loss-of-function analysis was then performed using TIMELESS-targeting siRNA oligos followed by a whole-genome expression microarray and network analysis. We further tested the effect of TIMELESS down-regulation on cell proliferation rates of a breast and cervical cancer cell line, as suggested by the results of our network analysis.

**Results:**

TIMELESS was found to be frequently overexpressed in different tumor types compared to normal controls. Elevated expression of TIMELESS was significantly associated with more advanced tumor stage and poorer breast cancer prognosis. We identified a cancer-relevant network of transcripts with altered expression following TIMELESS knockdown which contained many genes with known functions in cancer development and progression. Furthermore, we observed that TIMELESS knockdown significantly decreased cell proliferation rate.

**Conclusions:**

Our results suggest a potential role for TIMELESS in tumorigenesis, which warrants further investigation of TIMELESS expression as a potential biomarker of cancer susceptibility and prognostic outcome.

## Background

The circadian clock and cell cycle are two global regulatory systems that have pervasive effects on the behavior and physiology of eukaryotic cells. The 24-hour periodicity of the circadian rhythm, consisting of light and dark phases which coincide with the phases of the solar day, is maintained by a set of core circadian genes through a complex mechanism involving transcription-translational feedback loops [1,2]. The cell cycle is monitored by a sequence of molecular and biochemical events including a series of checkpoint mechanisms to ensure completion of biochemical reactions unique to each phase of the cell cycle prior to initiation of subsequent phases [3,4].

While these two regulatory systems involve distinct mechanisms, there is evidence that they are linked and interact at the gene, protein, and biochemical levels [5,6]. A recent study has indicated that one circadian regulator, *TIMELESS*, is also a core component of the cell cycle checkpoint system [7]. It regulates directly or indirectly the activity of autoregulatory components of the mammalian circadian core, including Clock, Per, and Cry proteins, associates with S phase replication checkpoint proteins Claspin and Tipin, and is required for the phosphorylation and activation of Chk1 by ATR and ATM-dependent Chk2-mediated signaling of DNA double strand breaks [8,9].

Although the connection between cancer and the cell cycle machinery that controls cell proliferation has been evident for some time, and there is mounting evidence to suggest that disruption of the circadian rhythm may increase susceptibility to certain malignancies [10-12], little is known about *TIMELESS*’s role in tumorigenesis. Our previous case–control study demonstrated significant genetic and epigenetic associations of *TIMELESS* and breast cancer risk [13]. A recent study has also shown that higher levels of *TIMELESS* expression in colorectal cancer tissue is associated with TNM stages III-IV and microsatellite instability [14]. In contrast, findings from another study point to the down-regulation of *TIMELESS* in hepatocellular carcinomas [15].

In the current study, we report our findings from the expression profiling analysis of *TIMELESS* in different tumor types using publically available online tools and microarray datasets, and a loss-of-function analysis using *TIMELESS*-targeting siRNA oligos followed by a whole-genome expression microarray and network analysis. We also tested one of the potential roles of *TIMELESS* suggested by our network analysis using a MTS assay and observed that *TIMELESS* knockdown decreased the proliferation rate of MCF7 breast cancer cells.

## Methods

### Data mining of TIMELESS expression in different tumor types

To explore whether *TIMELESS* expression is altered in different cancer types, we first performed a comprehensive search using the Oncomine 4.4 online database (https://www.oncomine.org; accessed on September 7, 2011) [16] for expression array comparisons involving tissues drawn from cancer patients and healthy controls. The keywords used were: Gene: “TIMELESS”; Analysis Type: “Cancer vs. Normal Analysis”. The search returned a total of 194 analyses conducted in 93 unique studies across various cancer types using different array platforms. Further details regarding tissue collection and the experimental protocol of each array are available in the Oncomine database, or from the original publications.

We then investigated whether aberrant *TIMELESS* expression was associated with tumor stage or prognostic outcome. We searched and analyzed publicly available microarray data sets containing tumor stage or clinical outcome information from the Gene Expression Omnibus (GEO) [17] and ArrayExpress databases (http://www.ebi.ac.uk/arrayexpress; accessed on September 8, 2011). The cervical cancer data set (GEO accession # GSE7803) contains gene expression data of normal cervical tissue, high-grade squamous intraepithelial lesions and invasive squamous cell carcinomas [18]. The ArrayExpress breast cancer data set (accession # E-TABM-276) examined gene expression in malignant breast tumor tissue, adjacent tissue exhibiting cystic changes, adjacent normal breast tissue and tissue drawn from healthy controls [19]. The prostate cancer data set GSE8511 includes tissue from benign prostate and localized and metastatic prostate tumor tissues [20], and GSE21034 contains samples from normal adjacent benign prostate and primary and metastatic prostate tumor tissues [21]. GSE2034 examined the association between gene expression in tissues drawn from primary breast cancer patients and their clinical outcomes [22]. The GOBO online tool (Gene Expression-Based Outcome for Breast Cancer Online co.bmc.lu.se/gobo), designed for prognostic validation of genes in a pooled breast cancer data set comprising 1881 cases from 11 public microarray data sets, was used to validate our analysis of the GSE2034 breast cancer data set [23].

### Cell culture and treatments

All experimental procedures were approved by the Institutional Review Board at Yale University and the National Cancer Institute. To determine *TIMELESS*’s role in tumorigenesis, we then performed an *in vitro* loss-of-function analysis using *TIMELESS*-targeting siRNA oligos followed by a whole-genome expression microarray. Human HeLa cells (American Type Culture Collection, Manassas, VA) were maintained in Dulbecco’s modified Eagle medium (Invitrogen, Carlsbad, CA) supplemented with 10% fetal bovine serum (Invitrogen) and 1% penicillin/streptomycin (Sigma-Aldrich, St. Louis, MO). Short interfering RNA (siRNA) oligonucleotides targeting exon 11 of *TIMELESS* (Ambion ID s17053; cat. no. 4392420) and a scrambled sequence negative control oligonucleotide were designed and manufactured by Ambion, Inc. (Ambion/Applied Biosystems). Each oligonucleotide was reverse-transfected in 12-well plates with ~10,000 cells/well at a final concentration of 10 nM using the Lipofectamine RNAiMAX transfection reagent (Invitrogen).

### RNA isolation and quantification

RNA was isolated using the RNA Mini Kit (Qiagen), with on-column DNA digestion, according to the protocols of the manufacturer for mammalian cells. RNA was quantified using a NanoDrop spectrophotometer (Thermo Scientific), and first-strand cDNA was synthesized using the AffinityScript cDNA Kit (Stratagene) with random ninemer primers. TIMELESS mRNA expression was measured by quantitative real-time PCR performed in duplicate using the Power SYBR Green PCR master mix (Applied Biosystems) and a standard thermal cycling procedure on an ABI 7500 instrument (Applied Biosystems). RNA quantity was normalized using *HPRT1*, and *TIMELESS* silencing was quantified using the 2^−ΔΔCt^ method.

### Genome-wide expression microarray

Gene expression differences in normal HeLa cells and those with reduced TIMELESS levels were examined by whole genome microarray (Agilent, Inc., 44 K chip, performed by MoGene, LC). RNA was isolated from biological replicates of each treatment condition (*TIMELESS*-targeting or scrambled negative control). Gene expression fold changes in *TIMELESS* knockdown cells relative to the mock siRNA-treated negative control were determined for each replicate. Samples with inadequate signal intensity (i.e., intensity < 50 in both the Cy3 and Cy5 channels), and transcripts with adjusted *P-*values greater than 0.05 in either biological replicate were discarded. To further reduce the number of false positive observations, and to enrich for biologically relevant expression changes, the remaining transcripts were defined as significantly differentially expressed only if they displayed a mean fold change in expression of at least |2|.

### Pathway-based network analysis

We then interrogated the differentially expressed transcripts for network and functional interrelatedness using the Ingenuity Pathway Analysis software tool (Ingenuity Systems; http://www.ingenuity.com). The software uses an extensive database of functional interactions which are drawn from peer-reviewed publications and are manually maintained [24]. *P*-values for individual networks were obtained by comparing the likelihood of obtaining the same number of transcripts or greater in a random gene set as are actually present in the input set (i.e., the set of genes differentially expressed following *TIMELESS* knockdown) using a Fisher's exact test, based on the hypergeometric distribution. Our microarray data were uploaded to the Gene Expression Omnibus [17] database (http://www.ncbi.nlm.nih.gov/projects/geo/; accession # pending). The differential expression of several genes detected by the microarray was assessed and confirmed by quantitative real-time PCR. The primers used were designed in house and the sequences are provided in Additional file 1: Table S1.

### Cell proliferation assay

The results from our network analysis suggested us to further investigate *TIMELESS*’s potential role in cellular growth and proliferation. HeLa and MCF7 cells (American Type Culture Collection) were reverse transfected with siRNA oligos targeting *TIMELESS* and a scrambled sequence negative control in 96-well plates using the Lipofectamine RNAiMAX transfection reagent (Invitrogen). Cell proliferation was analyzed in triplicate at baseline, 24 hours, 48 hours, 72 hours, and 96 hours using the CellTiter 96® AQueous One Solution Cell Proliferation Assay (MTS) kit (Promega Corporation, Madison, WI) and the absorbance was measured using an Epoch microplate spectrophotometer (BioTek, Winooski, VT).

### Statistical analyses

Statistical analyses were performed using the SAS statistical software, version 9.2 (SAS Institute). Student t-tests and one-way ANOVA were applied to calculate differences in *TIMELESS* expression across different tumor stages, as well as differences in cell proliferation rate. The log-rank test was used to estimate the differences in survival between cancer patients with differing levels of *TIMELESS* expression. Due to the multiple comparisons inherent in our microarray analysis, adjustments were made to control for false discoveries using the Benjamini-Hochberg method, as previously described, to obtain a false discovery rate-adjusted *P*-value for each observation (referred to as the *Q*-value) [25].

## Results

### Overexpression of TIMELESS in different types of tumor tissues

**Figure 1:**
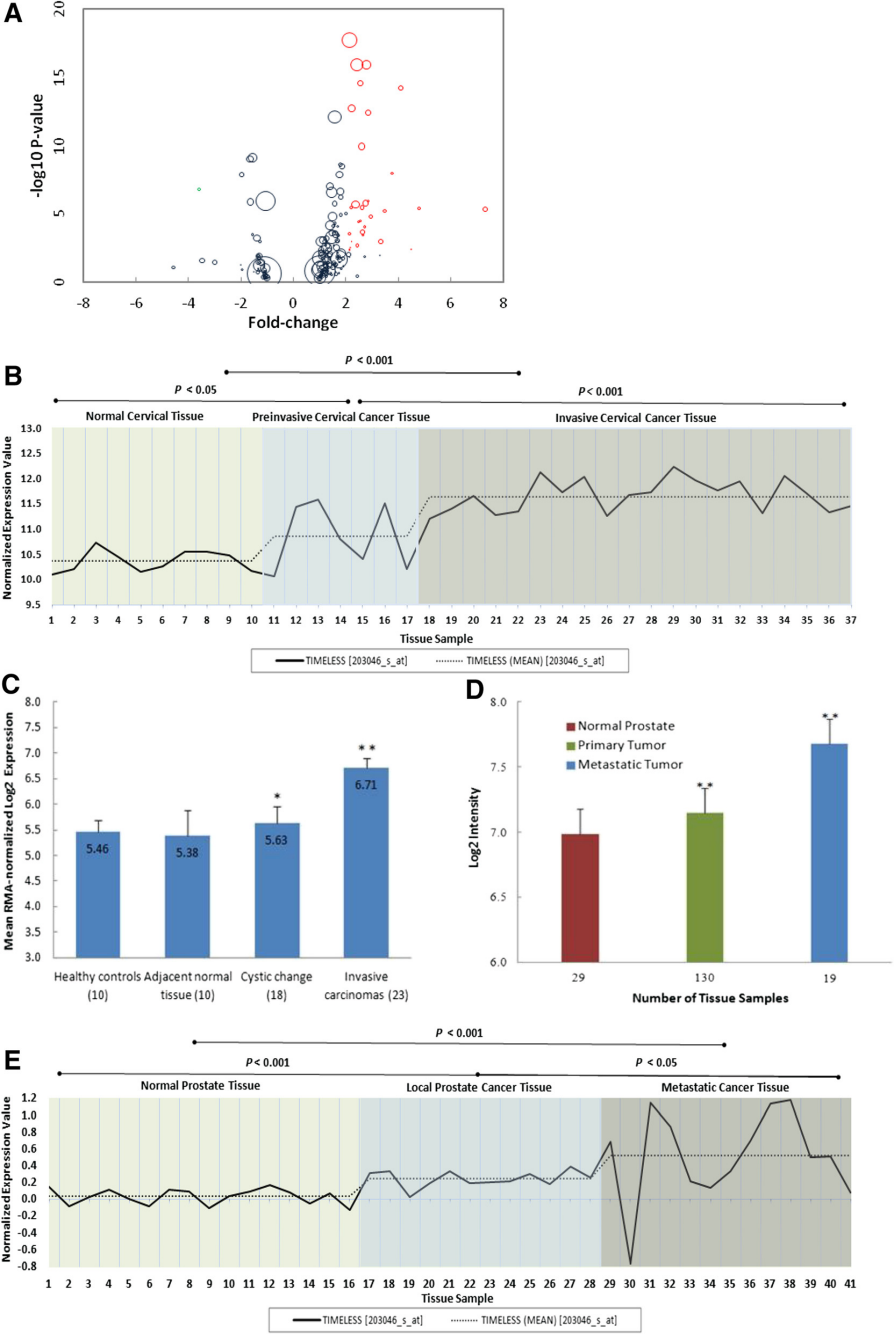
**Microarray data mining of *TIMELESS *expression in different tumor types. (A)***TIMELESS* expression in tumor tissues relative to controls from the Oncomine database. 31 out of 32 analyses showed higher *TIMELESS* expression while 1 analysis found lower *TIMELESS* expression. Analyses exhibiting *P*-values < 0.01 and fold-change values ≥ |2| are marked in red and green respectively. The size of each circle is scaled by the sample size of the corresponding analysis. **(B)***TIMELESS* expression in cervical cancer tissue versus preinvasive and normal tissue. Expression of *TIMELESS* in invasive cervical cancer tissues is significantly higher than in either normal or preinvasive tumor tissues. The original array data are from the Gene Expression Omnibus (accession # GSE7803). **(C)***TIMELESS* expression in breast tumor, adjacent tissues and tissues from healthy controls. *TIMELESS* expression in breast tissue from healthy controls and adjacent normal tissue was significantly lower than in invasive carcinomas or tissues exhibiting nonproliferative change (cystic change). Original array data is from the ArrayExpress database (accession # E-TABM-276). **(D)** and **(E)***TIMELESS* expression in prostate tumor and normal tissues. In normal prostate tissue, *TIMELESS* expression is significantly lower than in primary prostate tumor and metastatic tumor tissues. Metastatic tumor tissue exhibited the highest *TIMELESS* expression level compared to the other two groups. Original array data are from the Gene Expression Omnibus database (accession #'s GSE21034 and GSE8511).

Searching for “TIMELESS” expression in “cancer vs. normal” tissues in the Oncomine database returned a total of 194 analyses from 93 unique studies across various cancer types. 32 analyses in 20 unique studies were identified as statistically significant with *P*-values < 0.01 and fold change ≥ |2|. 31 out of 32 analyses exhibited increased *TIMELESS* expression in tumor relative to normal tissues while only one showed decreased expression (Additional file 1: Table S2). A volcano plot was generated using -log_10_ transformed *P*-values and the fold change of *TIMELESS* expression in tumor versus normal tissues extracted from each analysis. The size of each circle is proportional to the size of the analysis it corresponds to (Figure 1A). The plot indicates that *TIMELESS* expression is frequently elevated in tumor relative to normal tissues across multiple cancer types.

### Increased TIMELESS expression is associated with more advanced tumor stage and poorer breast cancer prognosis

To investigate whether *TIMELESS* expression is associated with tumor stage and clinical outcome, we analyzed five publicly available microarray data sets extracted from the GEO and ArrayExpress online databases: GSE7803 (cervical cancer), GSE21034 (prostate cancer), GSE8511 (prostate cancer), GSE2034 (breast cancer), and E-TABM-276 (breast cancer). We observed that *TIMELESS* expression in invasive cervical cancer tissue was significantly higher than in normal tissue (*P* < 0.001) and preinvasive cervical cancer tissue (*P* < 0.001) (Figure 1B). In the breast cancer study E-TABM-276, *TIMELESS* expression in breast tissue from healthy controls was significantly lower than in invasive carcinomas (*P* < 0.001) or tissues exhibiting cystic changes (*P* < 0.05). Likewise, *TIMELESS* expression in adjacent normal breast tissues was significantly lower than in either invasive carcinomas or tissues with cystic changes (*P* < 0.001 and *P* < 0.05, respectively) (Figure 1C). Similarly, in both of the two prostate cancer studies, significantly increased *TIMELESS* expression was observed in metastatic tumor tissue compared to primary prostate tumor tissue and benign tissue (Figure 1D and E).

**Figure 2:**
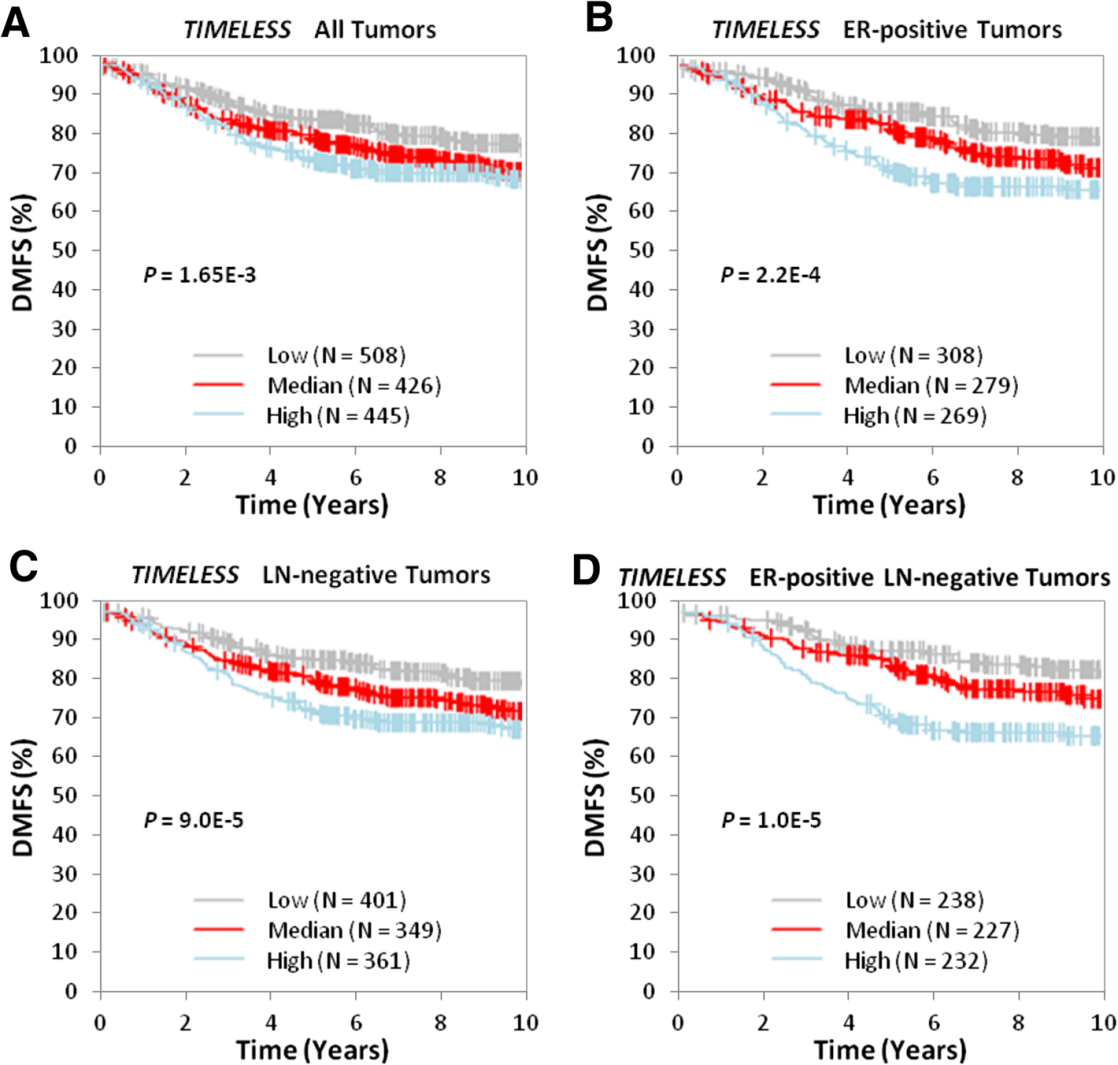
**Kaplan-Meier survival analysis of *TIMELESS *expression using the GOBO online tool, which comprises of pooled data from 1881 breast cancer cases from 11 public data sets.** Samples were stratified into tertiles based on *TIMELESS* expression level. The log-rank test was performed in all tumor samples as well as in different tumor subtypes using distant metastasis-free survival (DMFS) as the endpoint. High *TIMELESS* expression is significantly associated with lower DMFS over time among **(A)** all cases regardless of tumor ER- and LN-positivity (*P* = 1.65E-3), **(B)** cases with ER-positive tumors (*P* = 2.20E-4), **(C)** cases with LN-negative tumors (*P* = 9.00E-5), and **(D)** cases with ER-positive and LN-negative tumors (*P* = 1.00E-5).

Analyzing the lymph node-negative breast cancer data set of GSE2034, we found that patients with lower *TIMELESS* expression levels were more likely to have a higher rate of distant metastasis-free survival (DMFS) (*P* < 0.05). Interrogating *TIMELESS* expression using the GOBO database revealed similar results: increased *TIMELESS* expression was associated with lower DMFS rate not only in the general breast tumor population (*P* < 0.005), but also in tumor subtypes, including lymph node-negative (*P* < 0.001), ER-positive (*P* < 0.001), and lymph node- negative ER-positive (P < 0.001) breast tumors (Figure 2).

### Cancer-relevant network formed by TIMELESS-influenced genes

To explore *TIMELESS*’s potential functional significance in regulating cancer-relevant gene networks, we performed a loss-of-function analysis using *TIMELESS*-targeting siRNA oligos, followed by a whole genome expression microarray and subsequent network analysis. Prior to the microarray, *TIMELESS* knockdown was confirmed using quantitative RT-PCR. *TIMELESS* mRNA levels were reduced by more than 90% following knockdown (*P* < 0.01) (Additional file 2: Figure S1). In the array, 660 transcripts fit our significance criteria for differential expression following *TIMELESS* knockdown (*Q* < 0.05 and mean fold change ≥ |2|). Validation of differential expression was performed on nine genes using quantitative real-time PCR (Additional file 2: Figure S2). This gene set was examined for functional interrelatedness using the Ingenuity Pathway Analysis software tool. Cancer was identified as the top disease significantly associated with the input gene set, while cellular movement, development, and growth and proliferation were identified as the top three molecular and cellular functions.

**Figure 3:**
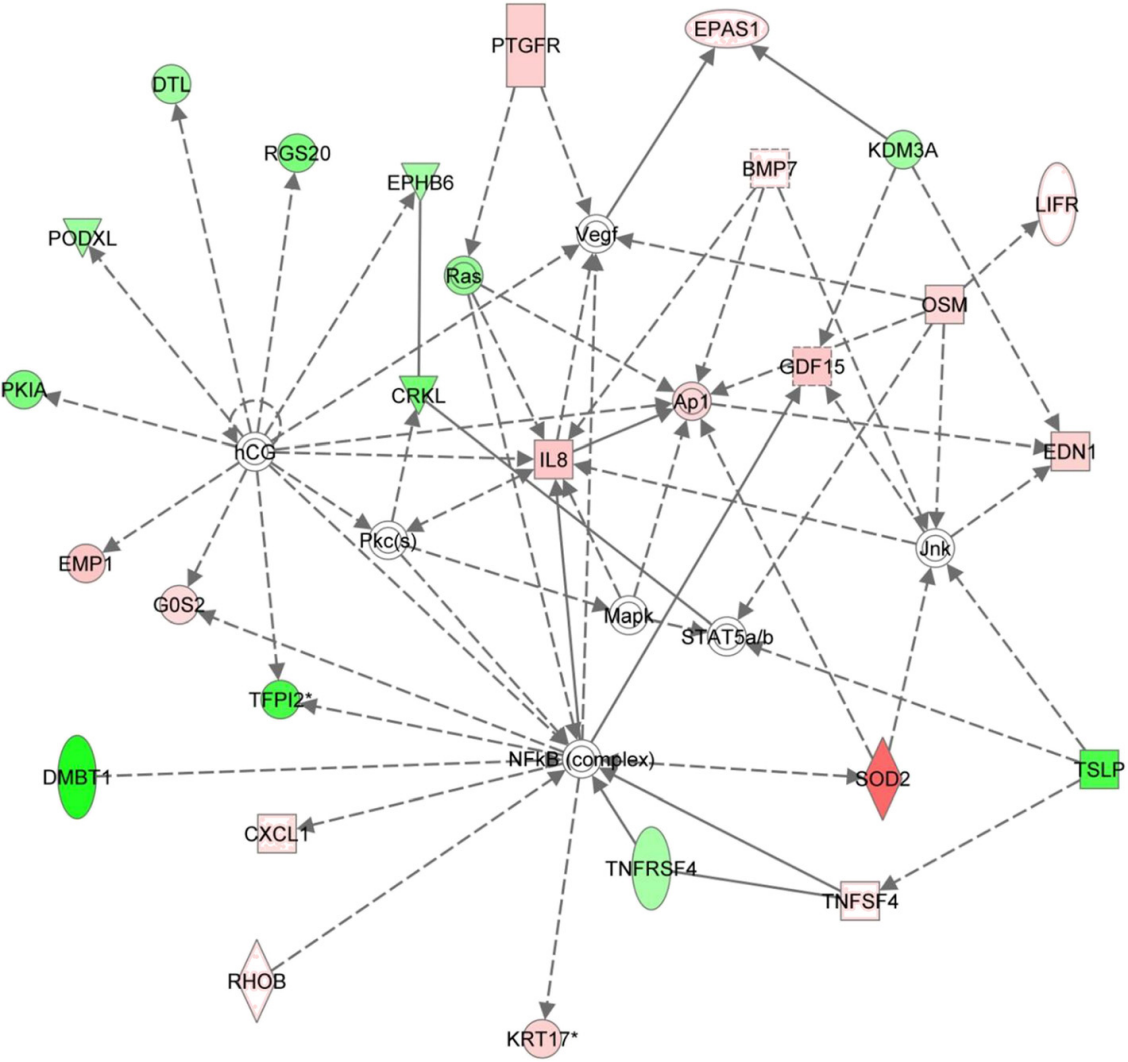
**The IPA-generated network most significantly associated with genes affected by *TIMELESS *knockdown.** According to the Ingenuity Pathway Analysis tool, the network is relevant to “cellular movement, immune cell trafficking, [and] gene expression”. Transcripts that were upregulated following *TIMELESS* knockdown are shaded in red, while transcripts that were downregulated are shaded in green, with color intensity signifying the relative magnitude of change. Each interaction is supported by at least one literature reference identified in the Ingenuity Pathway Knowledge Base, with solid lines representing direct interactions, and dashed lines representing indirect interactions.

Thirteen functional networks were identified as being significantly associated with the input gene set (*P* < 1.0E-10), the majority of which are cancer-related (Additional file 1: Table S3). The top functional network (*P* = 1.0E-32, Figure 3) formed by *TIMELESS*-affected genes was defined as having relevance for “cellular movement, immune cell trafficking, [and] gene expression”. Every one of the twenty-six genes within this top network has been reported to be involved in carcinogenesis or tumor progression. Among them, *CXCL1*[26], *EDN1 *[27], *EPAS1*[28,29], *GDP15*[30,31], *IL8*[32,33], *KRT17*[34,35], *CRKL*[36,37], *DTL*[38], *PTGFR*[39], *KDM3A*[40], *PODXL*[41], *RGS20*[42], and *TSLP*[43] are observed to be frequently overexpressed in cancer cells and are suggested to be involved in cancer development, tumor progression or poorer prognostic outcome. In contrast, *SOD2*[44,45], *RHOB*[46,47], *G0S2*[48], *EMP1*[49], *TNFRSF4*[50], *TNFSF4*[51], *DMBT1*[52,53], *LIFR*[54], *TFPI2*[55], and *EPHB6*[56] are frequently down-regulated in cancer and may be associated with tumor suppression or favorable prognostic outcome. A summary of the genes in this network, along with a brief description of relevant functions, *Q*-values and fold changes following *TIMELESS* knockdown, is presented in Table 1.

**Table 1:** **Molecules in the top (*P*** **= 1.0E-32) network of genes differentially expressed following *TIMELESS *** **knockdown**

**Symbol**	**RefSeq**	**Description**	**Fold change**	***Q*-Value**
*BMP7*	AL567265	Growth factor, may play a role in early development	2.41	5.2E-05
*CRKL*	NM_005207	Activates the RAS and JUN kinase signaling pathways and transform fibroblast in RAS-dependent fashion, candidate oncogene	-3.04	3.3E-06
*CXCL1*	NM_001511	Chemokine (C-X-C motif) ligand 1, regulates cell trafficking	2.10	1.4E-04
*DMBT1*	NM_007329	Plays a role in the interaction of tumor cells and the immune system, candidate tumor suppressor	-5.26	1.3E-12
*DTL*	NM_016448	Denticleless homolog (Drosophila), required for cell cycle control, DNA damage response and translesion DNA sythesis	-2.34	2.0E-11
*EDN1*	NM_001955	Endothelin 1, growth factor, involved in tumor progression	4.26	5.8E-32
*EMP1*	BC017854	Endothelia membrane protein 1	5.33	7.1E-13
*EPAS1*	NM_001430	Endothelia PAS domain protein 1, transcription factor, involved in the induction of genes regulated by oxygen	3.21	6.7E-11
*EPHB6*	NM_004445	EPH receptor B6, modulates cell adheson and migration, mediates numerous developmental processes, particularly in the nervous system.	-2.62	2.9E-05
*GOS2*	NM_015714	G0/G1 switch regulatory protein 2, potential oncogene	3.37	3.8E-09
*GDF15*	NM_004864	Growth differentiation factor 15, member of the transforming growth factor-beta superfamily, regulates tissue differentiation and maintenance	4.49	3.5E-12
*IL8*	NM_000584	Interleukin 8, cytokine, inhibits the proliferation of tumor cells	5.10	1.7E-11
*KDM3A*	NM_018433	May play a role in hormone-dependent transcription acivation and histone code, involved in spermatogenesis and obesity resistance	-2.13	1.5E-08
*KRT17*	NM_000422	Type I intermediate filament chain keratin 17, may be a marker for basal cell differentiation in complex epithelia	3.45	1.1E-07
*LIFR*	NM_002310	Leukemia inhibitory factor which is involved in cellular differentiation, proliferation and survival	2.77	2.7E-11
*OSM*	NM_020530	Cytokine, inhibits the proliferation of a number cell lines	3.23	1.5E-06
*PKIA*	NM_006823	Protein kinase inhibitor alpha	-3.30	7.2E-09
*PODXL*	NM_005397	Podocalyxin-like, involved in regulation of both adhesion and cell morphology and cancer progression	-2.47	3.5E-06
*PTGFR*	NM_000959	Prostaglandin F receptor	5.31	1.1E-08
*RGS20*	NM_170587	Regulation of G-protein signaling 20, accelarate transit through the cycle of GTP binding and hydrolysis and thereby accelerate signaling kinetics and termination	-3.34	3.4E-08
*RHOB*	NM_004040	Mediates apoptosis in neo plastically transformed cells after DNA damage, affects cell adhesion and growth factor signaling in transformed cells, involved in intracellular protein trafficking of a number of proteins	2.16	1.2E-04
*SOD2*	BC016934	Member of the iron/manganese superoxide dismutase family potential tumor suppressor	15.90	2.1E-14
*TFPI2*	ENST00000222543	Tissue factor pathway inhibitor 2, may play a role in the regulation of plasmin-mediated matrix remodeling	-5.19	4.9E-12
*TNFRSF4*	NM_003327	Tumor necrosis factor receptor superfamily, member 4, may suppresses apoptosis, plays a role in T cells-dependent B cell proliferation and differentiation	-2.09	8.6E-03
*TNFSF4*	NM_003326	Tumor necrosis factor (ligand) superfamily, member 4, directly mediates adhesion of activated T cells to vasular endothelial cells	2.18	6.9E-04
*TSLP*	NM_033035	Thymic stromal lymphopoietin, induces release of T cell-attracting chemokines from monocytes and enhances the maturation of CD11c(+) dendtritic cells	-4.68	8.0E-09

### TIMELESS knockdown decreases breast cancer cell proliferation rate

**Figure 4:**
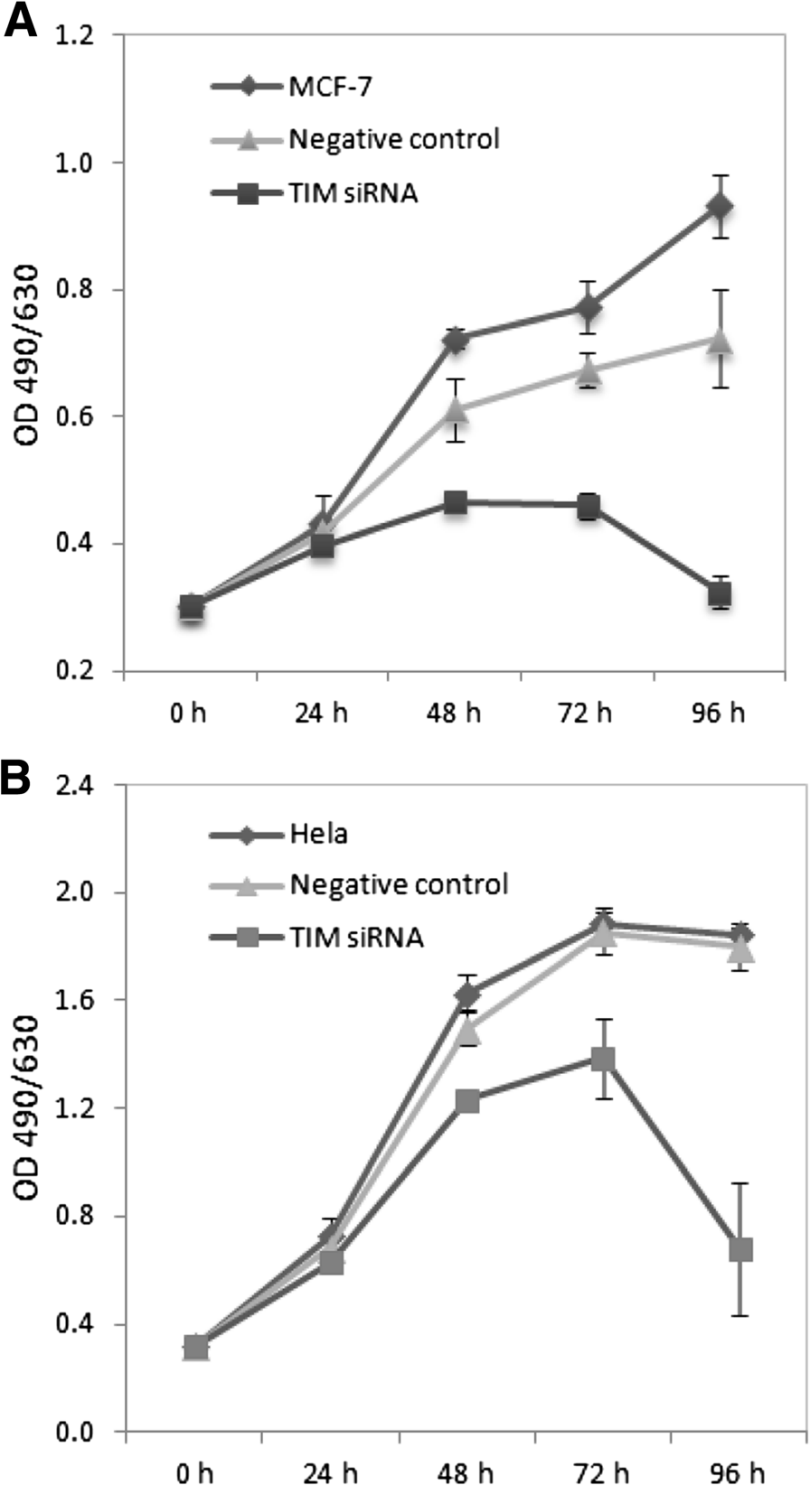
**MCF7 and HeLa cell proliferation rates were assessed at baseline, 24 hours, 48 hours, 72 hours, and 96 hours following transfection with a *TIMELESS *siRNA and a scrambled sequence negative control oligo. (A)** Transfection with *TIMELESS* siRNA in MCF7 cells slowed down cell proliferation compared to negative controls (*P* < 0.05); **(B)***TIMELESS* knockdown did not result in a significant reduction in cell proliferation rate in HeLa cells. Error bars represent standard deviations.

As suggested by the findings of our network analysis, we tested *TIMELESS*’s potential role in cellular growth and proliferation using a MTS assay. As shown in Figure 4, transfection with *TIMELESS*-targeting siRNA oligos significantly decreased MCF7 cell growth compared to untreated MCF7 cells (*P* < 0.05) and negative control cells (*P* < 0.05). A similar trend was observed with HeLa cells, but only a slight, yet not statistically significant, decrease in proliferation rate was observed compared to negative control cells (*P* = 0.156).

## Discussion

Since the hypothesis linking circadian disruption to increased breast cancer risk was first proposed twenty years ago, there have been many molecular epidemiologic studies implicating the tumorigenic importance of circadian variations, including genetic and epigenetic variations, and aberrant gene expression [10,57,58]. *TIMELESS*, which regulates directly or indirectly the activity of autoregulatory components of the mammalian circadian core, has been shown to play an essential role in the cell cycle checkpoint response [8,9]. As a potential molecular bridge between the cell cycle and the circadian regulatory systems, *TIMELESS* is also likely to play a significant role in tumorigenesis.

In our previous breast cancer case–control study, we found significant associations between two tagging SNPs in the *TIMELESS* gene and decreased breast cancer susceptibility. *TIMELESS* promoter hypomethylation in peripheral blood lymphocytes was also found to be significantly associated with later-stage breast cancer. In the current study, we observed that *TIMELESS* is frequently overexpressed in tumor relative to normal tissues in several cancer types, and that elevated expression of *TIMELESS* is significantly associated with later tumor stages and poorer breast cancer prognosis. Our findings also provide the first evidence suggesting the diagnostic and prognostic potential of *TIMELESS* in cancer.

Intriguingly, all 26 genes in the top IPA-generated network have been reported to be involved in cancer. *G0S2* (3.37-fold increase), which encodes a mitochondrial protein that specifically interacts with Bcl-2, is a proapoptotic factor, and its ectopic expression induces apoptosis in diverse human cancer cell lines in which endogenous *G0S2* is normally epigenetically silenced [48]. Similarly, RhoB (2.16-fold increase) is a well-characterized small GTPase that can inhibit cell proliferation, survival and invasion, and it is often down-regulated in cancer cells [47]. *EMP1* (5.33-fold increase) encodes a potential tumor suppressor that is associated with cellular proliferation and metastasis [49]. *DMBT1* (Deleted in malignant brain tumors 1 protein) (5.26-fold decrease) is a putative tumor suppressor gene frequently deleted in brain, gastrointestinal and lung cancers and down-regulated in breast cancer and prostate cancer [59]. Interestingly, Superoxide dismutase (*SOD2*), a probable tumor suppressor responsible for the destruction of superoxide free radicals [44], displayed a 15.9-fold increase in expression following *TIMELESS* knockdown. Additionally, Endothelin-1 (*EDN1*) (4.26-fold increase) encodes a growth factor that is frequently produced by cancer cells and plays a key role in cell growth, differentiation, apoptosis, and tumorigenesis [27]. Bone Morphogenetic protein 7 (*BMP7*) (2.41-fold increase), also known as osteogenic protein 1 (OP-1), encodes a multifunctional growth factor belonging to the TGF-β superfamily. Elevated BMP7 levels are reported to be correlated with the depth of colorectal tumor invasion, liver metastasis and cancer-related death [60], as well as the levels of estrogen and progesterone receptor, both of which are important markers for breast cancer prognosis and therapy [61]. Similarly, *GDF15* (4.49-fold increase), which encodes another member of the TGF-β superfamily, was reported to exert proapoptotic and anti-tumorigenic functions on colorectal, prostate, and breast cancer cells *in vitro* and on colon and blioblastoma tumors *in vivo*[62]. *IL8* (5.1-fold increase) has also been reported to have functions in the regulation of angiogenesis, cell growth and survival, leukocyte infiltration, and modification of immune responses [63]. These data suggest that loss of *TIMELESS* expression has the potential to influence a set of cancer-relevant genes, although most of these genes showing altered expression may not interact directly with *TIMELESS*. However, without further mechanistic investigations, it is not possible to identify whether these transcripts are direct or indirect targets of *TIMELESS*.

Timeless, together with its constitutive binding partner, Tipin, functions as a replisome-associated protein which interacts with components of the endogenous replication fork complex [64]. Moreover, siRNA-mediated *TIMELESS* down-regulation attenuates DNA replication efficiency [64]. Consistent with this observation, we observed a significant decrease in MCF7 cell proliferation after *TIMELESS* knockdown. However, we found only a slight but non-significant decrease in cell proliferation in HeLa cells following *TIMELESS* knockdown. This latter observation is consistent with the finding that *TIMELESS* down-regulation did not have a significant effect on cell proliferation in HeLa cells previously reported by Masai et al. [65]. As a recent study conducted by Engelen et al. revealed elevated *TIMELESS* expression in tissues undergoing active proliferation, the implication is that increased *TIMELESS* expression may be a characteristic of all highly proliferative cells, rather than one exclusive to cancer tissues. However, this relationship does not necessarily diminish the significance of *TIMELESS* in cancer simply because heightened cellular proliferation can be an important driver of the cancerous state. Even if *TIMELESS* expression is elevated as a result of, rather than a precursor to, heightened proliferation, *TIMELESS* expression may represent a natural response to abnormal proliferative rates and its potential physiological significance in cancer cannot be discounted. Further mechanistic studies are needed to investigate the precise role of *TIMELESS* on cellular growth and proliferation in different cancer types, as well as the capacity of *TIMELESS* to influence other potentially cancer-relevant pathways, including cell motility, invasiveness, and DNA damage response.

Although initial screening found a similar anti-proliferative response to a second siRNA, only the siRNA that conferred the greater phenotypic effect was chosen for subsequent assays. Given the inherent difficulty in controlling for off-target effects in any knockdown experiment performed using a single siRNA, the results presented here should be subjected to independent validation with use of a second siRNA. Furthermore, there is evidence to suggest that the anti-proliferative response observed from *TIMELESS* silencing could be partly attributable to apoptosis. It is evident that proliferation of transfected cells plateaus between the 48 hour and 72 hour time points and decreases thereafter, marking a period of gradual cell death. The degree to which silencing of *TIMELESS* elicits an apoptotic response should be the subject of a future investigation.

## Conclusions

In summary, these findings, although preliminary, support the findings from our previous breast cancer case–control study, and provide further evidence of the link between *TIMELESS* and carcinogenesis. The expression profiling analysis of the tissue-specific microarray data suggests that *TIMELESS* is frequently overexpressed in various types of tumor tissues, and elevated *TIMELESS* expression is associated with advanced tumor stage and poorer breast cancer prognosis. These data, in conjunction with the findings from the network analysis and the cell proliferation assay, suggest *TIMELESS* may be involved in the tumorigenic process. However, further mechanistic investigations are warranted to further elucidate the precise role of *TIMELESS* in tumorigenesis, and to help in the development of targeted therapeutic strategies.

## Competing interest

The authors declare that they have no competing interest.

## Authors’ contributions

YYM was responsible for performing database searches, analyzing microarray data, carrying out cell proliferation assays, and preparing the first manuscript draft. AF carried out the initial cell culture experiments and aided in manuscript preparation. DL helped to optimize conditions required for TIMELESS knockdown. YZ aided in experimental design and manuscript preparation. TZ and KC helped with manuscript preparation. All authors have read and approved the final manuscript.

## Pre-publication history

The pre-publication history for this paper can be accessed here:

http://www.biomedcentral.com/1471-2407/13/498/prepub

## Supplementary Material

Additional file 1: Table S1 Sequences of primers used for quantitative real-time PCR. **Table S2**: Details of the 32 analyses of TIMELESS expression in tumor compared to normal tissues when filtered by *P*-value < 0.01 and fold change ≥ |2| (Oncomine). **Table S3**: Details of the networks identified by the IPA software as significantly associated with the transcripts differentially expressed following *TIMELESS* knockdown. Click here for file

Additional file 2: Figure S1 *TIMELESS* knockdown confirmation in two biological duplicate populations of HeLa cells by real-time qPCR. **Figure S2**: Real-time qPCR confirmation of selected genes with differential expression following *TIMELESS* knockdown detected by the microarray analysis. Click here for file
