# Supplementary material for: Potential cancer-related role of circadian gene TIMELESS suggested by expression profiling and in vitro analyses
Source: BMC Cancer. 2013 Oct 25;13:498. doi: 10.1186/1471-2407-13-498 (PMC3924353; doi:10.1186/1471-2407-13-498)
Supplement: Additional file 1: Table S1 — Sequences of primers used for quantitative real-time PCR. Table S2: Details of the 32 analyses of TIMELESS expression in tumor compared to normal tissues when filtered by P-value < 0.01 and fold change ≥ |2| (Oncomine). Table S3: Details of the networks identified by the IPA software as significantly associated with the transcripts differentially expressed following TIMELESS knockdown. [file 1471-2407-13-498-S1.doc]

| **Supplementary Table 1.** Sequences of primers used for quantitative real-time PCR. | | |
| --- | --- | --- |
| **Genes** | **Forward primer (5’–3’)** | **Reverse primer (5’–3’)** |
| *TIMELESS* | GCAACAGGCATTCTCGATTT | TTTAGTTTCGTAGGTTGTGAAGG |
| *AR* | ACCATGTTTTGCCCATTGCA | GGTACTTCTGTTTCCCTTCAGC |
| *BNIP3L* | GAAAACATTCCACCCAAGGA | CCAAAGCCAAAACATGAGAAA |
| *CCND3* | TGGATGCTGGAGGTATGTGA | GCACAGTTTTTCGATGGTCA |
| *CDK6* | GCCCGCATCTATAGTTTCCA | TCAACATCTGAACTTCCACGA |
| *DMBT1* | CACTCAGGCCAGAAAGTTGG | CACATTGGCATCATTGGTGT |
| *EDN1* | CCAGAAACAGCAGTCTTAGGC | CCATACGGAACAACGTGCT |
| *EMP1* | ATTGCCAATGTCTGGTTGGT | CTTGAGGGCATCTTCACTGG |
| *GDF15* | CCAGCCTGGGCAACACTC | GGGGGCGCTCCTAGTAAA |
| *SOD2* | CTCCGGTTTTGGGGTATCTG | GGCTGTAACATCTCCCTTGG |

**Supplementary Table 2.** Details of the 32 analyses of TIMELESS expression in tumor compared to normal tissues when filtered by *P*-value < 0.01 and fold change > |2| (Oncomine).

**Supplementary Table 3.** Details of the networks identified by the IPA software as significantly associated with the transcripts differentially expressed following *TIMELESS* knockdown.

| **ID** | **Top Functions** | **Focus Molecules** | ***P*-value** |
| --- | --- | --- | --- |
| 1 | Cellular Movement, Immune Cell Trafficking, Gene Expression | 26 | 1.0E-32 |
| 2 | Cellular Growth and Proliferation, Cancer, Cellular Development | 25 | 1.0E-30 |
| 3 | Cellular Movement, Cellular Growth and Proliferation, Cell Death | 21 | 1.0E-23 |
| 4 | Cancer, Reproductive System Development and Function, Cell Morphology | 18 | 1.0E-18 |
| 5 | Gene Expression, Cell Cycle, Cellular Development | 18 | 1.0E-18 |
| 6 | Gene Expression, Hematological System Development and Function, Inflammatory Response | 16 | 1.0E-15 |
| 7 | Cellular Movement, Tissue Development, Cellular Development | 15 | 1.0E-14 |
| 8 | Gene Expression, Cell Cycle, Cell Death | 15 | 1.0E-14 |
| 9 | Cell Cycle, Cellular Growth and Proliferation, Gene Expression | 15 | 1.0E-14 |
| 10 | Cellular Growth and Proliferation, Connective Tissue Development and Function, Cell Death | 14 | 1.0E-12 |
| 11 | Cell Death, DNA Replication, Recombination, and Repair, Cell Cycle | 13 | 1.0E-11 |
| 12 | Genetic Disorder, Skeletal and Muscular Disorders, Tissue Morphology | 13 | 1.0E-11 |
| 13 | Gene Expression, Infectious Disease, Endocrine System Disorders | 12 | 1.0E-10 |
