# Supplementary material for: Potential cancer-related role of circadian gene TIMELESS suggested by expression profiling and in vitro analyses
Source: BMC Cancer. 2013 Oct 25;13:498. doi: 10.1186/1471-2407-13-498 (PMC3924353; doi:10.1186/1471-2407-13-498)

**Supplementary Figure 1.** *TIMELESS* knockdown confirmation in two biological duplicate populations of Hela cells by real-time qPCR.


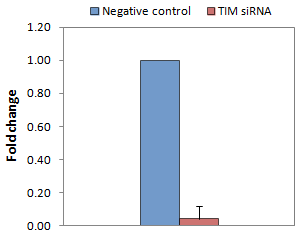
**Supplementary Figure 2.** Real-time PCR confirmation of selected genes with differential expression following *TIMELESS* knockdown detected by the microarray analysis.


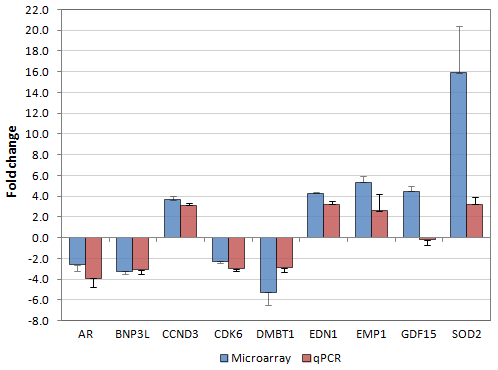

Supplement: Additional file 2: Figure S1 — TIMELESS knockdown confirmation in two biological duplicate populations of HeLa cells by real-time qPCR. Figure S2: Real-time qPCR confirmation of selected genes with differential expression following TIMELESS knockdown detected by the microarray analysis. [file 1471-2407-13-498-S2.docx]
